# Preoperative Radiotherapy in Elderly Patients With Locally Advanced Rectal Cancer: A Retrospective Cohort Study

**DOI:** 10.7759/cureus.92942

**Published:** 2025-09-22

**Authors:** Jacob F Wahba, Mohab Husien, Bogdan Paun, Gregory Knight, Mohamed Husein, Darin Gopaul

**Affiliations:** 1 Faculty of Health Sciences, Wilfrid Laurier University, Waterloo, CAN; 2 Faculty of Health Sciences, McMaster University, Hamilton, CAN; 3 Department of Surgery, Grand River Hospital, Kitchener, CAN; 4 Department of Oncology, Grand River Regional Cancer Centre, Kitchener, CAN; 5 Radiation Oncology, Grand River Regional Cancer Centre, Kitchener, CAN

**Keywords:** geriatric oncology, locally advanced rectal cancer, neoadjuvant chemoradiotherapy, neoadjuvant radiation treatment, oncological outcomes, rectal cancer, short-course radiotherapy, survival analysis

## Abstract

Purpose

To explore the outcomes of elderly patients (≥75 years) with locally advanced rectal cancer (LARC) treated with either short-course radiotherapy (SCRT) or chemoradiotherapy (CRT) at a regional cancer center.

Methods

A retrospective chart review was conducted for patients aged ≥75 with biopsy-confirmed rectal adenocarcinoma treated with either SCRT or CRT between January 2017 and June 2020. Patients were excluded if they had metastatic disease, recurrent cancer, or received palliative radiotherapy without surgical intent. SCRT consisted of 25 Gy in five fractions, with surgery performed either immediately or after a delay of six to eight weeks. CRT consisted of 50.4 Gy in 28 fractions with concurrent capecitabine, followed by delayed surgery. Outcomes assessed included surgical margin status, pathological complete response (pCR), local recurrence, distant metastasis (DM), overall survival (OS), and cancer-specific survival (CSS).

Results

A total of 46 patients met the inclusion criteria (SCRT: 34; CRT: 12). Radiotherapy was completed in 34 patients (100%) in the SCRT group and 11 patients (92%) in the CRT group. Surgery was performed in 28 (82%) of the SCRT patients and 11 (92%) of CRT patients. R0 resection was achieved in 86% (n=24) of SCRT and 100% (n=12) of CRT patients. No pathologic complete responses were observed. Local recurrence was observed in 2 SCRT patients (5.9%), and no recurrences occurred in the CRT group. Distant metastases developed in 3 SCRT (8.8%) and 2 CRT (16.7%) patients. With a median follow-up of 24 months, OS was 73.5% (SCRT) vs. 75% (CRT), and CSS was 73.5% (SCRT) vs. 91.7% (CRT), with no statistically significant differences.

Conclusion

Both SCRT and CRT were well tolerated and achieved high R0 resection rates with low recurrence in elderly patients with LARC. CRT showed slight trends toward greater nodal downstaging, while SCRT offered comparable survival with fewer treatment-related complications and greater flexibility in surgical timing.

## Introduction

Colorectal cancer is the fourth most diagnosed cancer in Canada in 2024 [1]. Older patients often carry a higher burden of comorbidities and frailty, which may impact their ability to tolerate standard treatment [2,3]. Preoperative chemoradiotherapy (CRT) for patients with locally advanced rectal cancer (LARC) is associated with increased tumor downstaging, increased rate of negative surgical margins, and reduced local recurrence [4,5]. However, in elderly patients, CRT is associated with increased treatment-related toxicity, raising questions about its tolerability and impact on quality of life [6]. These issues can contribute to treatment interruptions, early discontinuation of treatment, and reduced compliance, potentially leading to adverse effects and worsened oncologic outcomes. Short-course radiotherapy (SCRT), typically delivered as 25 Gy in five fractions over one week, is a well-established alternative to chemoradiotherapy, offering comparable oncological outcomes while significantly decreasing treatment duration [7,8].

Comparative studies in elderly patients remain limited, and many clinical trials exclude older adults or fail to report age-stratified outcomes. This retrospective study aims to explore clinical outcomes between SCRT and CRT in patients aged 75 years and older with LARC.

## Materials and methods

A retrospective chart review was conducted at the Waterloo Region Health Network (WRHN) for patients aged 75 years and older with biopsy-confirmed rectal adenocarcinoma treated with either SCRT or long-course CRT between January 2017 and June 2020 [9]. The study period of January 2017 to June 2020 was chosen due to a policy shift in mid-2020, due to the COVID-19 pandemic, which resulted in a large decrease in the use of CRT and SCRT and an increase in the adoption of the Rectal Cancer and Preoperative Induction therapy followed by Dedicated Operation (RAPIDO) approach. The inclusion criteria were patients staged as cT2-T4 and/or node-positive (cN+) and patients with no evidence of metastatic disease (stage IV) at presentation. Patients were excluded if they received neoadjuvant chemotherapy alone or if they were not considered surgical candidates and underwent palliative radiotherapy only.

Patients were stratified into three age groups: 75-79 (younger elderly), 80-89 (octogenarian), and ≥ 90 years (nonagenarian). The decision between SCRT and CRT was made at the discretion of the treating team based on factors such as age and comorbidities. SCRT consisted of 25 Gy in five fractions over one week. The immediate surgery group had surgery within 7 days. For the delayed surgery group, the surgery was performed approximately six to eight weeks after SCRT. CRT consisted of 50.4 Gy in 28 fractions with concurrent capecitabine (825 mg/m² orally twice daily on radiotherapy days), with surgery scheduled 6-8 weeks post-treatment. Postoperative management included no further chemotherapy, capecitabine alone, or FOLFOX, at the discretion of the treating medical oncologist. Selection was influenced by patient-related factors (age and comorbidities), tolerance of neoadjuvant therapy, and pathological risk factors such as nodal involvement or positive surgical margins.

All patients underwent initial staging with colonoscopy, CT of the chest/abdomen/pelvis (CT CAP), and pelvic MRI, unless contraindicated. Clinical staging followed the American Joint Committee on Cancer (AJCC) 8th edition [10]. Formal geriatric assessments were not routinely performed or documented during the study period. Surgical details, pathological findings, and oncologic outcomes were obtained from the electronic medical record. Pathologic complete response (pCR) was defined as ypT0N0.

Resection margins were considered negative (R0) if the tumor was >1 mm from the inked margin. Local recurrence was defined as any pelvic recurrence, detected by imaging and/or biopsy confirmation. Distant metastasis (DM) was detected by cross-sectional imaging and confirmed by biopsy when feasible. Overall survival (OS) was measured from the start of CRT until death from any cause or the date of last follow-up. Cancer-specific survival (CSS) was measured from the start of CRT until death from a cancer-related cause or the date of last follow-up. Cumulative incidence was used to estimate local recurrence rate (LRR) and DM, and the Kaplan-Meier method was used to assess OS. Statistical comparisons between treatment groups and age cohorts were performed using chi-square tests for categorical variables, with Fisher’s exact test applied when any category had fewer than five patients. Continuous variables were compared using t-tests. All statistical analyses were performed using R (version 4.5.1; The R Project for Statistical Computing) [11]. This study was approved by the Waterloo Wellington Research Ethics Board.

## Results

Cohort assembly

Of the 410 patients who received neoadjuvant radiotherapy for rectal cancer between January 2017 and June 2020, 73 were aged 75 or older. After excluding patients with recurrent cancer (n = 6), stage IV cancer (n = 13), or palliative radiotherapy/ non-surgical treatment (n = 8), a total of 46 patients met the inclusion criteria. Thirty-four patients received SCRT, and 12 received CRT.

Baseline characteristics

Patients and tumor characteristics are summarized in Table 1. Patients in the SCRT group were slightly older (median 82 years) compared to CRT (median 79 years). This difference was statistically significant (p = 0.03). The CRT group consisted mainly of younger elderly (67% aged 75-79), while SCRT was more often used in patients over 80, including two patients over 90 years old. No statistically significant differences were observed between groups in sex (p = 0.86), tumor stage (p = 0.48), clinical node stage (p = 0.21), or tumor level (p = 0.23).

**Table 1:** Baseline Characteristics of Elderly Patients with Locally Advanced Rectal Cancer by Treatment Group (SCRT vs. CRT) SCRT: short-course radiotherapy; CRT: chemoradiotherapy; M: male; F: female; χ²: chi-square test; t: independent samples t-test; FET: Fisher’s exact test

Characteristic	SCRT (n=34)	CRT (n=12)	Test Statistic (χ²/t/FET)	p-value
Median Age, years (range)	82.1 (75-92)	79.0 (75-85)	t = 2.2416	0.03
Sex M/F – no. (%)	18 (52.9)/16 (47.1)	6 (50.0)/6 (50.0)	χ²=0.0307	0.861
Age Group – no. (%)			FET	0.082
• 75–79	10 (29.4)	8 (66.7)		
• 80–89	22 (64.7)	4 (33.3)		
• 90+	2 (5.9)	0 (0.0)		
Clinical Tumor Stage – no. (%)			FET	0.477
• cT1/T2	5 (14.7)	0 (0.0)		
• cT2	0 (0.0)	0 (0.0)		
• cT2/Early cT3	7 (20.6)	2 (16.7)		
• cT3	13 (38.2)	6 (50.0)		
• cT3/Possible T4	6 (17.6)	1 (8.3)		
• cT4	2 (5.8)	2 (16.7)		
• Unknown	1 (3.0)	1 (8.3)		
Clinical Node Stage – no. (%)			FET	0.211
• N0	21 (61.7)	5 (41.7)		
• N1	8 (23.5)	6 (50.0)		
• N2	4 (11.8)	0 (0.0)		
• Unknown	1 (2.9)	1 (8.3)		
Distance from Anal Verge - no. (%)			FET	0.227
• <5 cm	12 (35.3)	2 (26.7)		
• 5–10 cm	19 (55.9)	7 (58.3)		
• >10 cm	2 (5.9)	1 (8.3)		
• Unknown	1 (2.9)	2 (16.7)		

Treatment delivery and surgery

All SCRT patients (34/34) completed radiotherapy. In the CRT group, 11/12 (92%) patients completing all 28 fractions in the CRT group (Table 2). The one patient that did not complete CRT died during treatment, representing the only treatment-related mortality (febrile neutropenia and sepsis) in the cohort. 

**Table 2:** Treatment and Surgical Outcomes in Elderly Rectal Cancer Patients by Preoperative Treatment Group SCRT: short-course radiotherapy; CRT: chemoradiotherapy; LAR: low anterior resection; APR: abdominoperineal resection; TAMIS: transanal minimally invasive surgery; χ²: chi-square test; t: independent samples t-test; FET: Fisher’s exact test

Variable	SCRT (n=34)	CRT (n=12)	Test Statistic (χ²/t/FET)	p-value
Completed Radiotherapy - no. (%)	34 (100)	11 (91.6)	FET	0.261
Surgery Performed - no. (%)	28 (82.4)	11 (91.6)	FET	0.657
Surgery Timing - no. (%)			FET	0.001
• Immediate Surgery	17 (60.7)	0 (0.0)		
• Delayed Surgery	11 (39.3)	11 (100)		
Surgery Type - no. (%)			FET	0.127
• LAR	16 (57.1)	4 (36.4)		
• APR	7 (25.0)	6 (54.5)		
• Hartmann’s	4 (14.3)	0 (0.0)		
• TAMIS	1 (3.6)	0 (0.0)		
• Unknown	0 (0.0)	1 (9.1)		
Differentiation Grade - no. (%)			FET	0.255
• Well-Differentiated	7 (25.0)	3 (27.3)		
• Moderately Differentiated	17 (60.7)	4 (36.4)		
• Poorly Differentiated	1 (3.6)	0 (0.0)		
• Unknown	3 (10.7)	4 (36.4)		
Pathologic T Stage - no. (%)			FET	0.622
• ypT0	0 (0.0)	0 (0.0)		
• ypT1	4 (14.3)	0 (0.0)		
• ypT2	6 (21.4)	3 (27.3)		
• ypT3	11 (39.3)	5 (45.5)		
• ypT4	5 (17.9)	1 (9.1)		
• Unknown	2 (7.1)	2 (18.2)		
Pathologic N Stage - no. (%)			FET	0.243
• ypN0	14 (50.0)	7 (72.7)		
• ypN1	8 (28.6)	1 (9.1)		
• ypN2	4 (14.3)	0 (0.0)		
• Unknown	2 (7.1)	2 (18.2)		
Resection Margin (R0) - no. (%)	24 (85.7)	11 (100)	FET	0.309

Surgery was performed in 28 SCRT and 11 CRT patients. Surgical timing differed significantly: all CRT patients had delayed surgery (6-8 weeks post-CRT), while 61% of SCRT patients proceeded to immediate surgery within 7 days, and the remainder had delayed surgery. Nineteen patients (49%) had a permanent colostomy. In total, LAR was the most frequent (16 SCRT, 4 CRT), followed by APR (7 SCRT, 6 CRT) and Hartmann’s procedure (4 SCRT). SCRT patients underwent a broader variety of operations, including local excision (TAMIS), which was not used in CRT. Six patients in the SCRT group did not undergo surgery. Two patients of those patients who developed liver metastases were managed with radiotherapy, and both died after. One patient declined surgery, and the remaining three were managed non-surgically without any further treatments. Of these three, two survived.

Pathologic findings

No patient achieved a pathologic complete response (ypT0N0). Major pathologic response (defined as ypT0-1N0) was observed in 11% (3/28) of patients in the SCRT group and 0% (0/11) in the CRT group. Most patients had residual T3 tumors; ypT3 was observed in 11 SCRT and 5 CRT patients. Node negative pathology was more common following CRT (73%) compared to SCRT (50%), but differences were not statistically significant. R0 resection was achieved in 89% of SCRT and all CRT patients (p = 0.3). Median surgical margin distance was similar between groups (SCRT: 11.0 mm vs CRT: 9.96 mm). Three SCRT patients had positive resection margins, with surgical margins ≤1 mm (two patients in the immediate surgery group and one patient in the delayed group). One patient experienced postoperative ileus and vomiting but remained cancer-free. A second patient had ileus postoperatively and was alive without recurrence at 6 months. The third patient, staged as cT1/T2N0 on pre-treatment imaging, underwent local excision with TAMIS. The final pathology showed a positive margin, and the patient later developed a local recurrence 23 months after surgery.

Age-stratified outcomes

All age-stratified outcomes are shown in Table 3. Treatment type varied significantly by age group (p = 0.001). Among the younger elderly patients, the majority received CRT (n=12) with 11 patients completing treatment. In contrast, most patients in the octogenarian group received SCRT (n=22), and both nonagenarian patients were treated with SCRT. All patients in the octogenarian and nonagenarian groups completed radiotherapy. Surgical resection was performed in the majority across all age groups (89% 75-79 vs. 81% 80-89 vs. 100% 90+; p-value = 0.5), and R0 resection rates remained high. No differences in recurrence, survival, or complications were observed between age groups. However, both patients over 90 died at 3 and 32 months, respectively.

**Table 3:** Oncologic Outcomes by Age Group in Elderly Patients with Locally Advanced Rectal Cancer Treated with Preoperative Radiotherapy pCR: pathological complete response; χ²: chi-square test; t: independent samples t-test; FET: Fisher’s exact test; N/A: not applicable

Outcome	75–79 (n=18)	80–89 (n=26)	90+ (n=2)	Test Statistic (χ²/t/FET)	p-value
Treatment Type (SCRT/CRT)	6 (33.3)/ 12 (66.7)	22 (84.6)/ 4 (15.4)	2 (100)/0 (0.0)	FET	0.001
Completed Radiotherapy (%)	17 (94.4)	26 (100)	2 (100)	FET	0.437
Surgery Performed (%)	17 (88.9)	21 (80.8)	2 (100)	FET	0.53
pCR Achieved (%)	0 (0.0)	0 (0.0)	0 (0.0)	N/A	N/A
R0 Resection (%)	16 (94.1)	19 (90.5)	2 (100)	FET	1
Alive at Last Follow-Up (%)	13 (72.2)	19 (73.1)	0 (0.0)	FET	0.13
Median Follow-Up (months)	25	21.8	N/A	t = 0.4392	0.663
Local Recurrence (%)	1 (5.6)	1 (3.8)	0 (0.0)	FET	1
Distant Metastasis (%)	3 (16.7)	2 (7.7)	0 (0.0)	FET	0.514

Postoperative complications

Twelve patients experienced postoperative complications, including ileus, hypotension, wound dehiscence, abscess, and renal dysfunction. One SCRT patient had multiple postoperative issues, including anastomotic leak, bowel dysfunction, and renal failure, though this did not appear to impact long-term survival. Another patient developed hemorrhagic wound dehiscence following SCRT and delayed APR, though they too remained recurrence-free.

Oncologic outcomes

**Figure 1:**
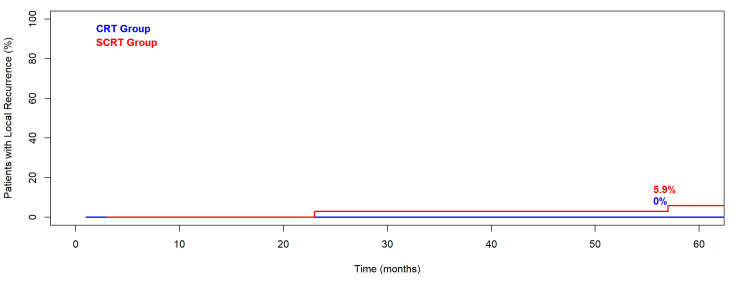
Cumulative Incidence of Local Recurrence by Treatment Type in Elderly Patients with Locally Advanced Rectal Cancer Treated with Neoadjuvant Radiotherapy CRT: chemoradiotherapy; SCRT: short-course radiotherapy

**Figure 2:**
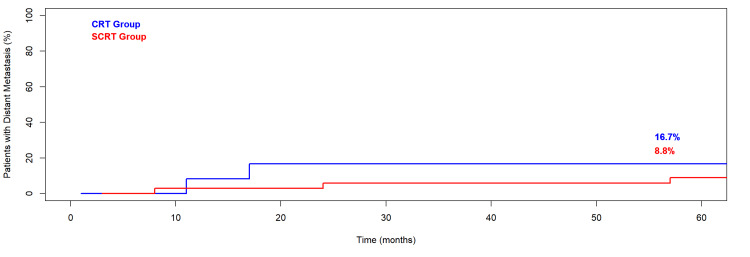
Cumulative Incidence of Distant Metastasis in Elderly Patients with Locally Advanced Rectal Cancer Treated with Neoadjuvant Radiotherapy CRT: chemoradiotherapy; SCRT: short-course radiotherapy

Two patients in the SCRT group experienced local recurrence (5.9%), and five developed distant metastases - three after SCRT and two after CRT. No statistically significant difference was observed in local recurrence or metastasis by treatment group (local recurrence p = 0.4, distant metastasis p = 0.5). The cumulative incidence of both local recurrence and distant metastasis is shown in Figure 1 and Figure 2. All metastatic cases received salvage therapy, including chemotherapy, partial hepatectomy, or radiotherapy. 

Multiple patients presented with significant comorbidities or synchronous malignancies that influenced the management of their rectal cancer. Two patients were unable to undergo MRI staging due to brain clips or a cardiac pacemaker, respectively. An 85-year-old patient with concurrent rectal and prostate cancer received CRT and LAR and remained alive without recurrence at 21 months. Another patient, with synchronously diagnosed rectal and lung cancers at 81 years of age, underwent SCRT and lung stereotactic body radiotherapy (SBRT) followed by immediate APR and remained disease-free at 44 months. An 88-year-old patient with synchronous rectal and cecal cancers received SCRT and underwent a delayed Hartmann’s procedure. This patient died after 23 months.

Overall and cancer-specific survival

**Figure 3:**
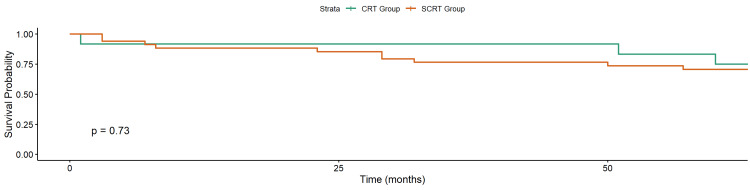
Kaplan–Meier Curve of Overall Survival in Elderly Patients with Locally Advanced Rectal Cancer Treated with Neoadjuvant Radiotherapy CRT: chemoradiotherapy; SCRT: short-course radiotherapy

**Figure 4:**
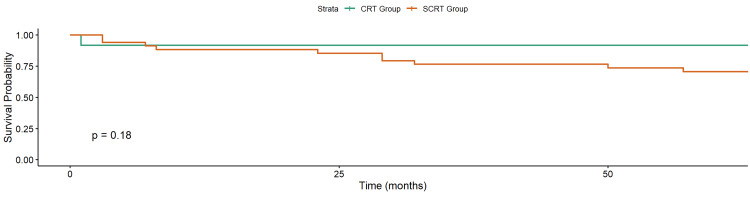
Kaplan–Meier of Cancer-Specific Survival in Elderly Rectal Cancer Patients Treated with Neoadjuvant Radiotherapy CRT: chemoradiotherapy; SCRT: short-course chemotherapy

At the last follow-up, 32 of 46 patients (69.6%) were alive, and 14 (30.4%) had died. OS was 73.5% in the SCRT group and 75% in the CRT group (p = 0.7; Figure 3). CSS was 73.5% in the SCRT group and 91.7% in the CRT group (p = 0.2; Figure 4).

## Discussion

This retrospective cohort study investigated the outcomes in elderly patients (≥75 years) with LARC treated with either SCRT or CRT at a single regional cancer center. Treatment completion was high in both groups, with all patients in the SCRT group and 92% in the CRT group completing radiotherapy. The potential toxicity of concurrent chemotherapy in elderly patients requires careful consideration. In our study, one treatment-related death and several complications occurred in the CRT group, underscoring the need to interpret chemotherapy-related risks separately from radiotherapy outcomes. Future studies should stratify toxicity data by treatment modality to better inform patient selection. In contrast, the completion of SCRT in all patients may be due to its favorable tolerability and shorter treatment duration, which may be especially beneficial for frail or comorbid elderly patients.

Surgical resection was completed in most patients across both treatment arms, with high rates of R0 resection in both groups (SCRT: 86%, CRT: 100%). SCRT patients had a broader range of surgical strategies, including local excision (TAMIS). pCR was not observed in either group, but nodal downstaging was slightly more pronounced in CRT-treated patients (ypN0 in 88% vs. 52%; p = 0.2), aligning with established evidence of CRT's impact on nodal downstaging [12]. However, this did not translate into statistically significant differences in recurrence or survival.

Although no patients in our cohort achieved a complete clinical or pathologic response, recent studies have examined non-operative (‘watch-and-wait’) strategies for patients demonstrating a complete response after neoadjuvant therapy [13]. In elderly patients, this approach has the potential to reduce surgical morbidity and preserve function; however, it remains controversial due to the risk of local recurrence and the requirement for intensive surveillance protocols [14]. While our data cannot directly address this question, it highlights the importance of future prospective studies to evaluate the safety and feasibility of surgery omission in carefully selected elderly patients.

The cumulative incidence of local recurrence (4.8%) and DM (11.9%) was low overall, with no significant difference between SCRT and CRT groups. Similarly, Kaplan-Meier survival analysis showed no significant difference in overall survival (p = 0.4), though numerically higher survival was observed in the CRT group (88.9 vs. 72.7%). This aligned with other meta-analyses [15]. The modest differences in distant metastasis and survival must be interpreted cautiously, given the small sample size and potential for treatment selection bias.

Treatment assignment differed significantly by age (p = 0.001), with CRT more common in patients aged 75-79 and SCRT used almost exclusively in patients ≥80. This is likely due to clinical concerns about the tolerability of chemoradiation in older adults with declining performance status or increased frailty [6,16,17]. Therefore, these analyses should be interpreted as descriptive only, providing insight into treatment patterns across the elderly spectrum rather than supporting inferential conclusions. Nonetheless, resection was completed in most patients across all age groups, including those over 90, and R0 resection rates remained high. However, both patients over age 90 died during follow-up, highlighting that patients over 90 should be carefully selected for surgery.

Several patients in our cohort had complex clinical profiles, including synchronous malignancies, significant comorbidities, or contraindications to standard imaging. These real-world scenarios show the detailed decision-making required in elderly oncology care and stress the adaptability of SCRT as a practical approach in patients who may not be ideal candidates for chemoradiation approaches [18].

Despite the valuable insights provided, the retrospective nature of this study introduces potential selection bias and limits causal inference. Treatment assignment was non-randomized and may have been influenced by unmeasured factors such as frailty, functional status, or patient preference. The relatively small sample size - particularly in the CRT group - reduces statistical power and may obscure clinically meaningful differences. Furthermore, variability in surgical techniques and follow-up duration across patients may affect the generalizability of these findings to broader populations. Another limitation is the absence of systematically recorded geriatric assessment data in the electronic medical record (EMR), which prevented us from analyzing this important factor. Future research should incorporate structured geriatric and cognitive tools, such as the Comprehensive Geriatric Assessment (CGA) test, to guide treatment selection and improve comparability across studies [19].

## Conclusions

In this retrospective analysis, both SCRT and CRT were associated with high rates of treatment completion, R0 resection, and low LRR in elderly patients with LARC. While CRT showed a slight trend toward greater nodal downstaging, SCRT offered comparable oncologic outcomes with fewer treatment-related complications and more options for surgical timing. These findings support the use of SCRT as a safe and effective alternative to CRT in appropriately selected older adults and emphasize the importance of individualized treatment strategies guided by geriatric assessment. Future prospective studies should prioritize inclusion of elderly patients and explore patient-centered outcomes such as functional preservation, quality of life, and treatment burden.
